# Endothelium-specific deletion of p62 causes organ fibrosis and cardiac dysfunction

**DOI:** 10.1186/s12967-024-04946-w

**Published:** 2024-02-16

**Authors:** Jing Feng, Yan Li, Yu Zhang, Shengnan Sun, Jian Sun, Quanlin Xu, Xingzhao Ji, Yi Liu, Qiang Wan

**Affiliations:** 1 Key Laboratory of Cell Metabolism in Medical and Health of Shandong Provincial Health Commission, Jinan Central Hospital, Shandong University, Jinan, 250021 Shandong China; 2 Qingdao Central Hospital, Shandong University, Qingdao, 266042 Shandong China; 3 Department of Pulmonary and Critical Care Medicine, The Second Hospital of Shandong University, Jinan, 250033 Shandong China; 4 Department of Pulmonary and Critical Care Medicine, Shandong Provincial Hospital, Shandong University, Jinan, 250021 Shandong China; 5 Department of Pulmonary and Critical Care Medicine, Shandong Provincial Hospital Affiliated to Shandong First Medical University, Jinan, 250021 Shandong China; 6 Shandong Key Laboratory of Infections Respiratory Disease, Medical Science and Technology Innovation Center, Shandong First Medical University & Shandong Academy of Medical Sciences, Jinan, 250021 Shandong China; 7 Key Laboratory of Cell Metabolism in Medical and Health of Shandong Provincial Health Commission, Central Hospital Affiliated to Shandong First Medical University, Jinan, 250021 Shandong China

**Keywords:** SQSTM1/p62, Endothelium cells, Fibrosis, Cardiac dysfunction, Fibronectin1, Lamininγ2

## Abstract

**Background:**

The autophagy adapter SQSTM1/p62 is crucial for maintaining homeostasis in various organs and cells due to its protein–protein interaction domains and involvement in diverse physiological and pathological processes. Vascular endothelium cells play a unique role in vascular biology and contribute to vascular health.

**Methods:**

Using the Cre-loxP system, we generated mice with endothelium cell-specific knockout of p62 mediated by Tek (Tek receptor tyrosine kinase)-cre to investigate the essential role of p62 in the endothelium. In vitro, we employed protein mass spectrometry and IPA to identify differentially expressed proteins upon knockdown of p62. Immunoprecipitation assays were conducted to demonstrate the interaction between p62 and FN1 or LAMC2 in human umbilical vein endothelium cells (HUVECs). Additionally, we identified the degradation pathway of FN1 and LAMC2 using the autophagy inhibitor 3-methyladenine (3-MA) or proteasome inhibitor MG132. Finally, the results of immunoprecipitation demonstrated that the interaction between p62 and LAMC2 was abolished in the PB1 truncation group of p62, while the interaction between p62 and FN1 was abolished in the UBA truncation group of p62.

**Results:**

Our findings revealed that p62 ^Endo^ mice exhibited heart, lung, and kidney fibrosis compared to littermate controls, accompanied by severe cardiac dysfunction. Immunoprecipitation assays provided evidence of p62 acting as an autophagy adapter in the autophagy-lysosome pathway for FN1 and LAMC2 degradation respectively through PB1 and UBA domain with these proteins rather than proteasome system.

**Conclusions:**

Our study demonstrates that defects in p62 within endothelium cells induce multi-organ fibrosis and cardiac dysfunction in mice. Our findings indicate that FN1 and LAMC2, as markers of (EndoMT), have detrimental effects on HUVECs and elucidate the autophagy-lysosome degradation mechanism of FN1 and LAMC2.

**Supplementary Information:**

The online version contains supplementary material available at 10.1186/s12967-024-04946-w.

## Introduction

P62, also known as SQSTM1, is a versatile scaffolding molecule weighing 62 kDa. It possesses multiple domains that facilitate interactions with various binding partners, ultimately regulating diverse signal transduction pathways. Notably, p62's ZZ domain, p38-binding sequence, TBS and KIR motif have been implicated in cell survival, inflammation, amino acid sensing, mitochondrial biogenesis, brown adipose tissue thermogenesis, and oxidative stress response [1–4]. As an autophagy adaptor, p62 primarily mediates autophagy through its Phox1 and Bem1p (PB1) domain, ubiquitin-associated (UBA) domain, and LC3-interacting region (LIR) [5]. Given its involvement in critical intracellular signaling pathways, the role of p62 in disease pathogenesis has garnered significant attention. Abnormal expression and activity of p62 have been implicated in the development and progression of various cancers and neurodegenerative diseases. Experimental evidence strongly supports the involvement of p62 in atherosclerosis, type II diabetes, and obesity. Particularly, whole-body deficiency of p62 in mice leads to prominent late-onset obesity and insulin resistance [6]. Mice lacking p62 demonstrate increased atherosclerotic plaque burden, accompanied by heightened macrophage inflammation, apoptosis, necrotic core formation, and plaque complexity [7]. These effects stem from p62-mediated protein aggregation, which hampers inflammasome activation and occurs independently of NRF2, ERK, p38MAPK, or NF-κB signaling pathway inhibition [8, 9]. Neuron-specific p62 deficient mice also exhibit an obese phenotype due to defective STAT3 activity, resulting in leptin resistance, hyperphagia, and mature-onset obesity [10]. In our previous study, we identified p62 as a crucial regulator of mtDNA expression machinery, with its effects mediated by p38-dependent upregulation of mitochondrial ribosomal protein L12 (MRPL12) in renal tubular epithelial cells (TECs) [11]. Notably, TEC-specific p62 conditional knockout (CKO) mice displayed evident kidney injuries at 5 months of age, characterized by oliguria and elevated serum creatinine and BUN levels, underscoring the essential role of p62 in maintaining TEC homeostasis.

Endothelium cells (ECs) play a crucial role in vascular biology and their dysfunction is strongly associated with the development and progression of various vascular diseases, including cardiovascular, pulmonary vascular, cerebrovascular, and diabetic vascular complications. Autophagy serves as a key regulator of EC homeostasis and function. Impaired autophagy in ECs leads to a notable loss of EC markers, such as E-cadherin, during the process of endothelium-to-mesenchymal transition (EndoMT) [12]. The interplay between autophagy and EndoMT is also evident in the progression of vascular-related diseases [13]. For instance, mice with endothelium cell-specific knockout of ATG16L1 exhibit increased susceptibility to cell death upon treatment with alpha-toxin [14]. Similarly, endothelium cell-specific knockout of ATG5 accelerates diabetic nephropathy and capillary rarefaction by exacerbating pathological glomerular alterations in mice with podocyte autophagy defects [15].

However, the precise changes that occur in endothelium cells upon p62 deficiency, as well as the impact on the cardiovascular system and potential non-autophagy functions of endothelium p62, remain unknown and warrant further investigation. Thus, in this study, we generated Tek receptor tyrosine kinase (Tek)-cre-mediated endothelium cell-specific p62 knockout mice using the Cre-loxP system to elucidate the critical role of p62 in the endothelium. Our findings revealed that endothelium-specific deletion of p62 resulted in multi-organ fibrosis and cardiac dysfunction. Furthermore, we investigated the underlying mechanism of fibrosis and demonstrated that p62 regulates FN1/LAMC2 degradation through the autophagolysosomal pathway. Evidence suggests a close relationship between autophagy levels and the development of organ fibrosis. Aberrant autophagy can be regarded as one of the pathogenic mechanisms of fibrosis. Targeting autophagy holds promise as an effective therapeutic target for organ fibrosis, providing insight into potential treatment strategies or clinical interventions. For example, the use of autophagy-enhancing drugs has been shown to promote the degradation of mutant SERPINA1/α1-antitrypsin Z and alleviate hepatic fibrosis [16]. Moreover, a specific inhibitor (AC-73) has been demonstrated to activate autophagy by targeting BSG/CD147 and alleviate chronic colitis-associated intestinal fibrosis induced by trinitrobenzenesulfonic acid [17]. In addition, blocking autophagic flow mediated by linc00941/lncIAPF-ELAVL1/HuR promotes fibroblast differentiation, providing new targets and effective therapeutic strategies for autophagy-based treatments for idiopathic pulmonary fibrosis [18]. Recent studies further confirm that chronic autophagy deficiency or impaired autophagic function may accelerate renal fibrosis [19]. LYC plays an important role in activating mitochondrial autophagy and improving renal fibrosis [20]. Therefore, theoretically, targeting autophagy mechanisms may contribute to the improvement of fibrosis development. This potential therapeutic strategy will promote the clinical translation for fibrotic diseases.

## Materials and methods

### Animals

Endothelium-specific p62 knockout mice (p62^Endo^) were generated by crossing mice positive for vascular endothelium-cadherin Cre recombinase (Tek-Cre) with mice carrying the p62 loxP/loxP floxed allele (p62^flox/flox^) (n = 5). Control groups consisted of Tek Cre-negative p62flox/flox littermates (p62^f/f^) (n = 5).

### Reagents

The following antibodies were used: rabbit polyclonal anti-SQSTM1 (Cat.No. 18420-1-AP), anti-FN1 (Cat.No. 15613-1-AP), anti-LAMC2 (Cat.No. 19698-1-AP), anti-BECN1 (Cat No. KHC1731) and anti-ACTIN (Cat.No. 23660-1-AP) obtained from Proteintech Group, as well as mouse monoclonal anti-αSMA (53–9760-82), anti-collagen IV (14-9871-82), and anti-Ecad (14-3249-82) purchased from Thermo Fisher Scientific.

### Transfection

Human umbilical vein endothelium cells (HUVECs) and HEK 293 T cells cultured in DMEM medium supplemented with 10% fetal bovine serum was used for transfection experiments. HUVECs and 293 T cell were passaged in 6-well plates with DMEM medium for the transfection studies. Transfection was performed using Lipofectamine 3000 transfection reagent (Invitrogen) and 25 nM of p62 siRNA (RIBBIO) according to the manufacturer's instructions. The cells were incubated for 6 h with the Lipofectamine-siRNA complex, followed by replacement of the medium with fresh experimental medium. The cells were then incubated for an additional 24 h. After incubation, the cells were harvested using RIPA buffer (containing PMSF, sodium vanadate, and protease inhibitor; Solarbio) for western blotting analysis.

### Immunofluorescence

Cells were inoculated onto a Petri dish containing a pre-placed coverslip. Once the cells reached near confluence, they were fixed with 4% paraformaldehyde for 20 min and washed three times with PBS for 5 min each. A 1:200 dilution of Triton X-100 in PBS was used for permeabilization, and the cells were incubated for 5–15 min. Following permeabilization, the cells were washed three times with PBS for 5 min each. Blocking was performed using 1% goat serum for 1 h, followed by washing with PBS for 5 min three times. The p62 fluorescent antibody was added, and the cells were incubated overnight at 4 °C in the dark. Subsequently, the second fluorescence antibody was added, and the cells were incubated at room temperature in the dark for 1 h. After three washes with PBS, DAPI was added dropwise, and fluorescence microscopy was used for visualization.

### Heterogeneous immunofluorescence double staining

Paraffin sections were deparaffinized and rehydrated. After Antigen repairing, circled for serum blocking using 10% serum from goat and 3%BSA from fetal bovine. Added the mixed reagents of the CD31 and p62 antibodies in a wet box for incubation at 4℃ overnight. Added secondary antibody and then counterstained nucleus by DAPI. Quenched tissue autofluorescence for Microscopy detection and images collected by Fluorescent Microscopy. DAPI glowed blue by UV excitation wavelength 330-380 nm and emission wavelength 420 nm; 488 glowed green by excitation wavelength 465–495 nm and emission wavelength 515-555 nm; CY3 glow red by excitation wavelength 510-560 nm and emission wavelength 590 nm.

### Real-time PCR

Total RNA was extracted from frozen kidneys using TRIzol (Ambion, LOT304308) according to the manufacturer’s instructions. RNA concentration was measured using a Nanodrop spectrophotometer. Complementary DNA (cDNA) was synthesized using the PrimeScript RT Reagent Kit (Perfect Real Time; Takara, Cat.RR047A). SYBR Green PCR kit (Accurate Biology, Lot.A4A2293) was used for gene expression quantification with 10 ng of cDNA. Primers for p62 were Fwd 5ʹ-GTGGTAGGAACCCGCTACAA-3ʹ and Rev 5ʹ-TGTGCGAGAAGCCCTCAGAC-3ʹ, while primers for FN1 were Fwd 5ʹ-TGTGAACATCCCTGACCTGC-3ʹ and Rev 5ʹ-CAGGCGCTGTTGTTTGTGAA-3ʹ. The primers for LAMC2 were Fwd 5ʹ-CCAAGGTGAGGAGCCAAGAG-3ʹ and Rev 5ʹ-TGAGCCTGTGAGTATCCCGA-3ʹ, and the primers for ACTIN were Fwd 5ʹ-CATGTACGTTGCTATCCAGGC-3ʹ and Rev 5ʹ-CTCCTTAATGTCACGCACGAT-3ʹ.

### Western blotting

Protein expression in harvested HUVECs was analyzed by SDS-PAGE and western blotting. Samples with equal protein concentrations, determined using the BCA Protein Assay (Beyotime, Cat. No. P0011), were mixed with sample buffer and heated at 100 °C for 5 min. The samples were loaded onto 5–20% SDS-PAGE gels. Separated protein lysates were transferred to PVDF membranes (Millipore, LOT0000185906) using the semi-dry method. After blocking with TBS-T (Tris-buffered saline [20 mM Tris–HCl, pH 7.6, 150 mM NaCl] containing 0.05% Tween 20) and 5% BSA, the membranes were incubated overnight at 4 °C with primary antibodies of interest diluted in TBS-T with 5% BSA. Following three washes, the membranes were incubated with HRP-conjugated secondary antibodies (Proteintech, Cat. No. PR30011) at a dilution of 1:1000 for 1 h at room temperature. Immunoreactive target bands were detected using an enhanced chemiluminescence (ECL) system (Tanon5200Multi).

### Co-immunoprecipitation assays

HUVECs were incubated for 24 h, washed three times with ice-cold PBS, and lysed using IP lysis buffer (Thermo Scientific, YA362594) supplemented with a protease inhibitor (Solarbio, Cat. No. P1260). The cell lysates were centrifuged at 14,000 rpm for 10 min, and the supernatants were collected. The supernatants were incubated overnight at 4 °C with an anti-p62 antibody (Proteintech, Cat.No.18420–1-AP) or rabbit IgG (Proteintech, Cat.No.30000-0-AP). Protein agarose beads were added and mixed at room temperature for 2 h, followed by centrifugation to collect the precipitated complex. The beads were washed four times with PBS. Finally, the precipitated proteins were eluted and denatured in 4 × SDS loading buffer and analyzed by western blotting. 293 T cells were separately transfected with pcDNA3.1, the overexpression plasmid of full-length p62 with a flag tag (HOMO-SQSTM1 -FLAG/His in plent), and plasmids with PB1 (delete amino acids 21–85) (HOMO-SQSTM1-△PB1-FLAG/His in plent) and UBA (delete amino acids 385–440) (HOMO-SQSTM1-△UBA-FLAG/His in plent) domains truncated. Cells were collected after 48 h, and the remaining procedures were the same as described above. Anti-flag antibody (Cell Signaling Technology, Cat.No.14793S) was used for truncation experiment.

### Flow cytometry for apoptosis

After the incubation period, HUVECs were harvested and washed with cold phosphate-buffered saline (PBS). A 1X annexin-binding buffer was prepared. A working solution of PI at a concentration of 100 µg/mL was prepared by diluting 5 µL of the 1 mg/mL PI stock solution. The washed cells were centrifuged again, the supernatant was discarded, and the cells were resuspended in 1X annexin-binding buffer. Cell density was determined, and the cells were diluted in 1X annexin-binding buffer to achieve a concentration of approximately 1 × 10^6^ cells/mL, with a final volume of 100 µL per assay. To each 100 µL of cell suspension, 5 µL of Alexa Fluor® 488 annexin V (Component A) and 1 µL of the 100 µg/mL PI working solution were added. The cells were incubated at room temperature for 15 min. Next, 400 µL of 1X annexin-binding buffer was added, gently mixed, and kept on ice. The stained cells were analyzed by flow cytometry, measuring the fluorescence emission at 530 nm.

### Wound healing assay

HUVECs were grown to confluency. A linear wound was created by scraping a pipette tip across the confluent cell layer. Cells were then washed twice to remove detached cells and debris. The size of the wounds was observed and measured at the indicated times.

### Echocardiogram

Transthoracic echocardiography was performed using a vevo3100LT system with an MS-400 transducer. Mice were anesthetized with 2% isoflurane inhalation and placed on a heated platform maintained at 37˚C. The heart rate was stabilized between 450–550 beats per minute before acquiring echocardiographic measurements. Two-dimensional B-mode imaging was used to visualize the left ventricle and the aortic outflow tract. A sample line was placed at the maximum cross-section of the left ventricle to guide the recording of serial M-mode echocardiographic images. Measurements including fraction shortening (FS), ejection fraction (EF), interventricular septum (IVS), left ventricular posterior wall thickness (LVPW), left ventricular mass (LVmass), left ventricular end-diastolic internal diameter (LVIDd), and left ventricular end-systolic internal diameter (LVIDs) were obtained from at least three distinct frames for each mouse.

### Transmission electron microscope

Discarded the culture medium of HUVEC and added pre-chilled pre-fixation solution at 4℃, then fix for 15 min. Gently scraped off the cells using a cell scraper. Centrifuged at 2000–3000 rpm for 10 min to pellet the cells at the bottom of the centrifuge tube. Rapidly removed the aorta and cut the tissue block into approximately 1.0 mm*1.0 mm*1.0 mm in size, immediately immersed it in pre-chilled 4% paraformaldehyde fixation solution for 2 h. After fixation, washed the fixed cells or tissue samples with phosphate buffer solution for 0.5 h at 4 °C. Fixed with 1% osmium tetroxide at 4 °C for 2 h, followed by washing with buffer solution. Performed gradient dehydration of the samples using ethanol after tissue dehydration. After tissue embedding with an embedding agent for 30 min, performed staining.

### Immunohistochemical staining (IHC)

After dissecting the lung tissue from mice, we fixed it with 4% paraformaldehyde. For dehydration, a general protocol involved gradually increasing concentrations of ethanol from low to high. Then, performed clearing with xylene, followed by tissue embedding in paraffin and sectioning. Baked the sections at 45 °C for 2 h, performed antigen retrieval using citrate buffer, blocked with goat serum, and incubated with Beclin1, FN1 and LAMC2 antibodies separately. Tissue sections were incubated with secondary antibody and visualize using DAB staining. Counterstaining with hematoxylin, dehydrated and cleared the sections, and mounted them with neutral mounting medium. Observed and captured images under a microscope.

### Statistical analysis

All data are presented as mean ± SEM. Statistical analyses were performed using Prism 8 software (GraphPad Software, San Diego, CA). Comparison between two groups were analyzed using t-test. A p-value < 0.05 was considered statistically significant.

## Results

### Tek-specific deletion of p62 causes cardiac dysfunction in vivo.

**Fig. 1:**
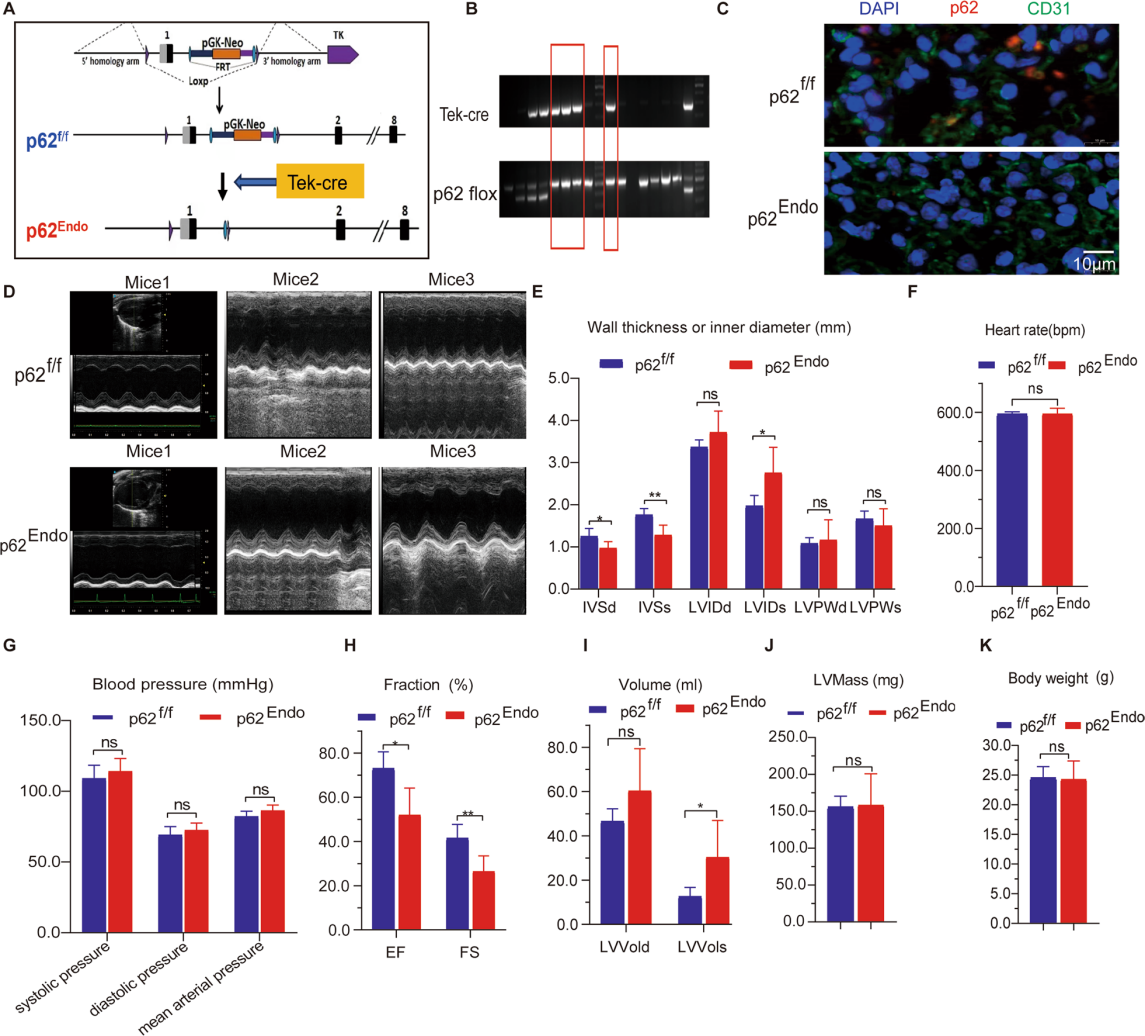
Endothelium knockout of p62 induces cardiac dysfunction in mice. **A** Generation of endothelium-specific knockout mice for p62. **B** Identification of genes in the tail of mice. Tek-Cre mutant: 300 bp.p62 mutant: 525 bp.Wild type:400 bp. Mice within the red frame were the p62^Endo^ mouse we needed. **C** The immunofluorescence co localization of endothelial markers CD31 (green) and p62 (red) in lung tissue. **D** Echocardiography analysis in mice. **E** Assessment of systolic and diastolic parameters including ventricular septal thickness, left ventricular diameter, and left ventricular posterior wall thickness in mice. **F** Measurement of heart rate in mice. **G** Evaluation of systolic, diastolic, and mean arterial pressure in mice. **H** Calculation of ejection fraction and shortening fraction in mice. **I** Analysis of left ventricular systolic and diastolic end volume in mice. **J** Determination of left ventricular mass in mice. **K** Monitoring of body weight changes in mice. * *p* < 0.05 and ** *p* < 0.01

Using the Cre-loxP system, we generated endothelium-specific p62 knockout mice mediated by Tek receptor tyrosine kinase (Tek)-cre. These mice had p62 inactivated specifically in endothelium cells (referred to as p62^Endo^), while p62 was not inactivated in control mice (p62^f/f^) (Fig. 1A). Genotype identification confirmed the successful generation of the p62^Endo^ mice (Fig. 1B). The immunofluorescence co localization of endothelial markers CD31 (green) and p62 (red) in lung tissue demonstrated the absence of p62 expression in the endothelium of p62^Endo^ mice (Fig. 1C).

Our investigation commenced with a meticulous observation of murine at 3 months, wherein no discernible disparities in survival rates were observed. Furthermore, we conducted ultrasonic echocardiography assessments, revealing an absence of cardiac insufficiency (Additional file 1: Fig. S1A). Subsequently, heart and lung tissue dissections were performed on the murine specimens, with both groups exhibiting no overt dissimilarities nor presenting any manifestations of fibrosis (Additional file 1: Fig. S1B, 1C). It was not until the mice reached the age of 12 months that respiratory distress, diminished locomotive activity, and deteriorated condition were manifested exclusively in p62 ^Endo^ mice. At 12 months of age, p62^Endo^ mice exhibited significant cardiac dysfunction (Fig. 1D). Echocardiography revealed systolic dysfunction, with a profound decrease in the interventricular septum (IVS) thickness during diastole and systole compared to control mice (p62^fl/fl^ versus p62^Endo^: IVS at end-diastole 1.255 ± 0.184 versus 0.9780 ± 0.148, P = 0.0303; IVS at end-systole: 1.764 ± 0.145 versus 1.284 ± 0.233, P = 0.0044) (Fig. 1E). The left ventricular internal diameter (LVID) increased significantly during systole but not during diastole in p62^Endo^ mice (p62^fl/fl^ versus p62^Endo^: left ventricular internal diameter at end-diastole 3.372 ± 0.167 versus 3.724 ± 0.499, P = 0.1735; left ventricular internal diameter at end-systole: 1.975 ± 0.25 versus 2.762 ± 0.603, P = 0.0273) (Fig. 1E). Left ventricular posterior wall thickness (LVPW) remained largely unchanged during diastole and systole in p62^Endo^ mice (p62^fl/fl^ versus p62^Endo^: LVPW at end-diastole 1.084 ± 0.134 versus 1.168 ± 0.476, P = 0.7140; LVPW at end-systole: 1.668 ± 0.186 versus 1.508 ± 0.398, P = 0.4392) (Fig. 1E). Heart rate (Fig. 1F) and blood pressure (Fig. 1G), including diastolic pressure, systolic pressure, and mean arterial pressure, did not differ significantly between the two groups. However, p62^Endo^ mice exhibited impaired systolic function, with a decrease in ejection fraction (EF) and fraction shortening (FS) compared to control mice (p62^fl/fl^ versus p62^Endo^: EF:73.11 versus 52.03, P = 0.0107; FS: 41.48 versus 26.43, P = 0.0073) (Fig. 1H). The left ventricular volume (LVVol) was not increased during diastole but showed a significant increase during systole in p62^Endo^ mice (p62^fl/fl^ versus p62^Endo^: 46.63 ± 5.61 versus 60.36 ± 19.01, P = 0.1601 during diastole; 12.62 ± 4.05 versus 30.36 ± 16.61, P = 0.0489 during systole) (Fig. 1I). Previous studies have demonstrated that p62 knockout can lead to an obese phenotype. We also took this into consideration and monitored the body weight of the mice from birth until 1 year of age. However, we did not observe any statistically significant differences in LV mass (Fig. 1J) and body weight (Fig. 1K) between the two groups.

### Tek-specific deletion of p62 causes cardiovascular fibrosis and inflammatory infiltration

**Fig. 2:**
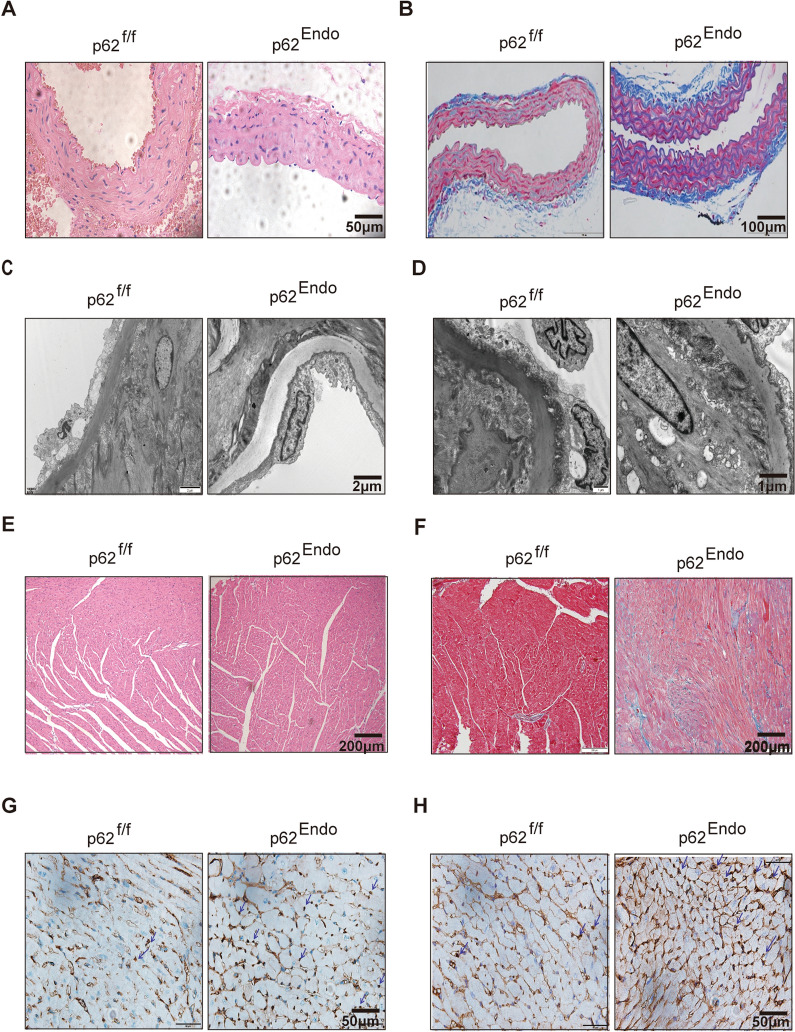
Endothelium knockout of p62 induces cardiovascular tissue fibrosis and inflammatory infiltration. **A** HE staining of the aorta. Scale bars = 50 μm. **B** Masson’s trichrome staining of the aorta. Scale bars = 100 μm. **C** Low magnification electron microscopy image of the mouse aorta. Scale bars = 2 μm. **D** High magnification electron microscopy image of the mouse aorta. Scale bars = 1 μm. **E** HE staining of mouse myocardium. Scale bars = 200 μm. **F** Masson’s trichrome staining of mouse myocardium. Scale bars = 200 μm. **G** CD68 staining of mouse cardiomyocytes. CD68 is a marker of macrophages. The arrow is pointing to macrophage. Scale bars = 50 μm. **H** LCA staining of myocardial cells in mice. LCA(Leukocyte Common Antigen) is a marker of lymphocytes. The arrow is pointing to lymphocyte. Scale bars = 50 μm

At 12 months of age, mice were sacrificed, and the aorta and heart were collected and fixed in 4% paraformaldehyde for staining. Additionally, a portion of the aorta was soaked in Glutaraldehyde for transmission electron microscopy. Histopathological examination of the organs using hematoxylin and eosin staining (HE staining) and Masson’s trichrome staining revealed significant fibrosis in the aorta of mice with Tek-specific deletion of p62 compared to control mice (Fig. 2A, B).

Furthermore, examination of murine aortic ultrastructure using transmission electron microscopy showed that the endothelium cells in the aorta of p62^Endo^ mice exhibited slight edema and formed gaps with the sub-endothelium layer. This observation was made through microscopic analysis under both low magnification (2 μm) (Fig. 2C) and high magnification (1 μm) (Fig. 2D). Although there was no significant alteration in the number of mitochondria, they appeared slightly swollen in p62^Endo^ mice.

Consistent with the results obtained from echocardiography, histopathological examination of the heart using HE staining revealed minor infarcts in p62^Endo^ mice compared to control mice (Fig. 2E). Masson’s trichrome staining of 12-month-old murine hearts demonstrated excessive collagen deposition in the p62^Endo^ group, indicating elevated cardiac fibrosis, a characteristic feature of pathological cardiac remodeling (Fig. 2F). Additionally, immunostaining for CD68 and LCA showed increased infiltration of macrophages and lymphocytes, respectively, in the myocardial tissue of p62^Endo^ mice (Fig. 2G, H). These findings collectively suggest that p62 in the adult cardiac endothelium plays an essential role in maintaining cardiac endothelium homeostasis.

### P62^Endo^ mice shows tissue fibrosis in the lung and kidney

**Fig. 3:**
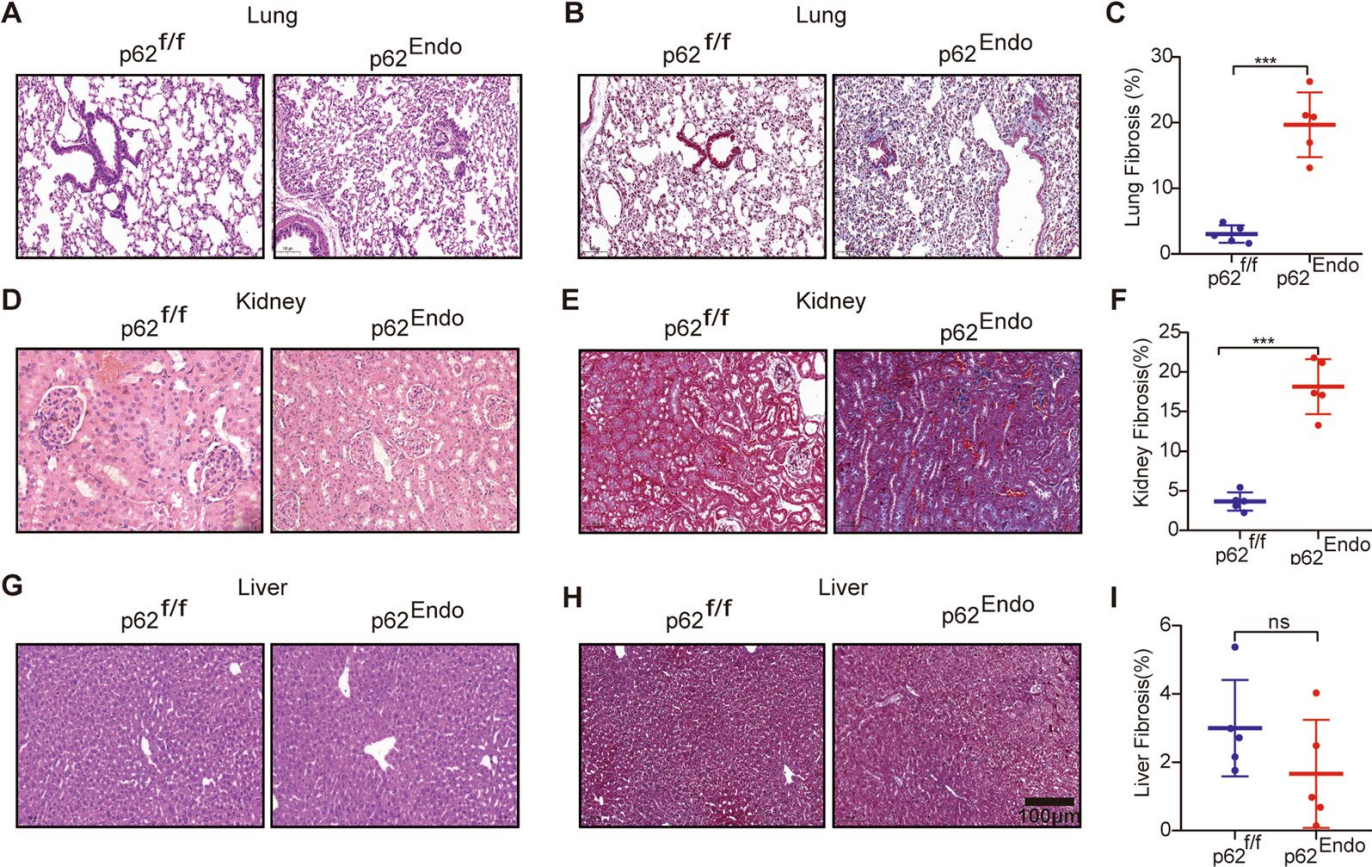
Endothelium knockout of p62 induces renal and pulmonary fibrosis. **A** HE staining of lung tissues. **B** Masson's trichrome staining of the lung. **C** Semi-quantitative analysis of Masson's trichrome staining in the lung. **D** HE staining of kidney tissues. **E** Masson's trichrome staining of the kidney. **F** Semi-quantitative analysis of Masson's trichrome staining in the kidney. **G** HE staining of liver tissues. **H** Masson's trichrome staining of the liver. **I** Semi-quantitative analysis of Masson's trichrome staining in the liver. *** *p* < 0.001. Scale bars = 100 μm

Given the important role of endothelium cells in multiple systems, we investigated whether the knockout of p62 in endothelium cells would have an impact on other tissues. To explore this, we performed HE and Masson staining on the lungs, kidneys, and livers. HE staining revealed thickened alveolar septa and glomerulosclerosis in p62^Endo^ mice (Fig. 3A). Masson staining showed obvious fibrosis accompanied by collagen deposition in the lung (Fig. 3B, C). No obvious abnormalities in HE staining of renal pathology (Fig. 3D) while accompanied by collagen deposition in kidney by Masson staining (Fig. 3E, F) with Tek-specific deletion of p62 compared to control mice. However, no significant pathological changes were observed in liver tissue (Fig. 3G) and no significant fibrotic changes were observed in the liver tissue of p62^Endo^ mice (Fig. 3H, I).

### Different effects of p62^Endo^ mice on kidney function, liver function, and blood lipids

**Fig. 4:**
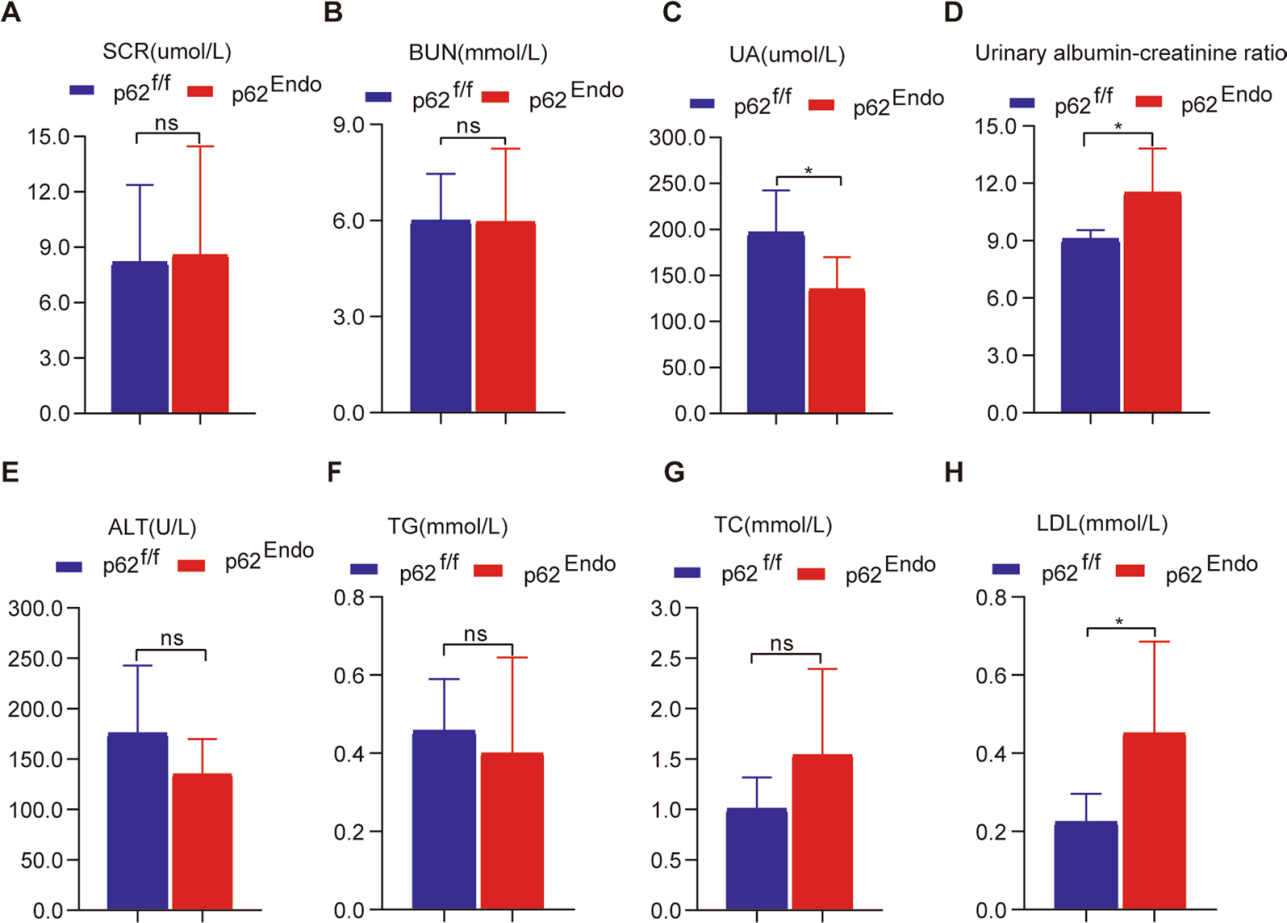
Differential effects of endothelium knockout of p62 on liver and kidney function, and blood lipids in mice. **A** Levels of serum creatinine (SCR) in mice. **B** Levels of serum urea nitrogen (BUN) in mice. **C** Levels of serum uric acid (UA) in mice. **D** Urine protein creatinine ratio in mice. **E** Levels of serum alanine aminotransferase (ALT) in mice. **F** Levels of serum triglycerides (TG) in mice. **G** Levels of serum total cholesterol (TC) in mice. **H** Levels of low-density lipoprotein cholesterol (LDL) in mouse serum. * *p* < 0.05

Serum and urine samples were collected from the mice at the age of 12 months to assess kidney function, liver function, blood lipid levels, and other indicators. There was no significant difference in serum creatinine (p62^fl/fl^ versus p62^Endo^: 5.99 ± 1.47 versus 5.96 ± 2.28, P = 0.98) (Fig. 4A) and serum urea nitrogen levels (p62^fl/fl^ versus p62^Endo^: 8.18 ± 4.2 versus 8.58 ± 5.8, P = 0.90) (Fig. 4B) between the two groups. However, uric acid levels were significantly decreased in p62^Endo^ mice (p62^fl/fl^ versus p62^Endo^: 196.5 ± 45.9 versus 135.1 ± 34.8, P = 0.04) (Fig. 4C). The urinary albumin-creatinine ratio, an indicator of renal dysfunction, was elevated in the p62^Endo^ group (p62^fl/fl^ versus p62^Endo^: 9.080 versus 11.52, P = 0.0483) (Fig. 4D).

No obvious hepatic dysfunction was observed in the p62^Endo^ group, as indicated by the levels of alanine aminotransferase (ALT) (p62^fl/fl^ versus p62^Endo^: 175.6 ± 67.1 versus 135.1 ± 34.8, P = 0.2649) (Fig. 4E). Mild dyslipidemia was observed in p62^Endo^ mice, with no significant differences detected in triglyceride (TG) levels (p62^fl/fl^ versus p62^Endo^: 0.46 ± 0.13 versus 0.4 ± 0.24, P = 0.6532) (Fig. 4F) and total cholesterol (TCH) levels (p62^fl/fl^ versus p62^Endo^: 1.0 ± 0.3 versus 1.5 ± 0.85, P = 0.2229) (Fig. 4G). However, LDL levels were significantly increased in the p62^Endo^ group compared to the p62f/f group (p62^fl/fl^ versus p62^Endo^: 0.22 ± 0.07 versus 0.45 ± 0.23, P = 0.002) (Fig. 4H), indicating that p62 in endothelium cells plays an important role in maintaining lipid homeostasis.

### Protein mass spectrometry of HUVEC p62 knockdown (KD) and negative control (NC) reveal enrichment in the extracellular space and matrix

**Fig. 5:**
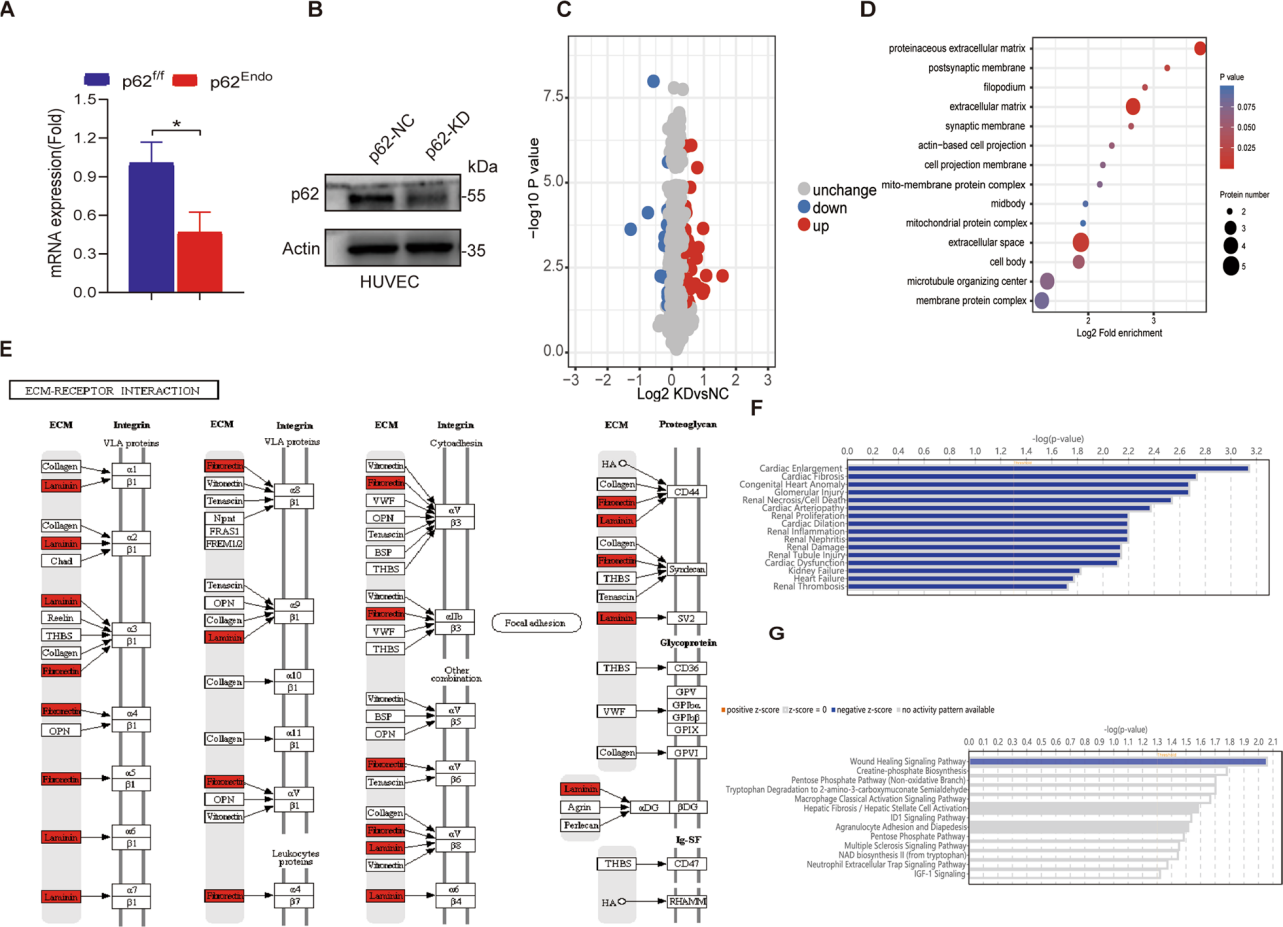
Omics and IPA analysis of HUVEC p62 knockdown reveals enrichment of extracellular matrix. **A** Validation of p62 knockdown efficiency using quantitative real-time PCR (qRT-PCR). **B** Verification of p62 knockdown efficiency by Western Blot. **C** Volcano distribution map of differential genes. **D** Bubble diagram depicting cellular components identified through mass spectrometry analysis. **E** Pathway enrichment analysis highlighting the involvement of the extracellular matrix pathway. **F** Diseases enriched according to IPA analysis. **G** Enriched pathways identified by IPA analysis

To gain insights into the phenotypic changes in HUVECs and murine tissues with p62 knockdown, we conducted a quantitative proteomic analysis using TMT labeling, high-performance liquid chromatography, and mass spectrometry-based techniques. We confirmed the efficiency of p62 knockdown through PCR (Fig. 5A) and western blotting (Fig. 5B). A total of 6225.0 proteins were identified, out of which 5643.0 proteins had quantitative information. Using a threshold of 1.2-fold change and a statistical significance threshold of t-test p-value < 0.05, we identified 51 up-regulated proteins and 14 down-regulated proteins in the KD versus NC comparison group (Fig. 5C).

Based on these findings, we performed systematic bioinformatics analysis, including functional annotation, functional classification, functional enrichment, and cluster analysis for all differentially expressed proteins. We observed a significant enrichment of differentially expressed proteins in certain functional categories, particularly in the extracellular space and matrix, as indicated by the distribution bubble map from the cluster analysis (Fig. 5D). Notably, FN1 and LAMC2 were major proteins involved in the extracellular matrix receptor interaction pathway (Fig. 5E).

Using Ingenuity Pathway Analysis, we found that the differentially expressed proteins were enriched in cardiac-related diseases based on protein mass spectrometry, including cardiac enlargement, cardiac dysfunction, and heart failure (Fig. 5F). Importantly, the wound-healing pathway was found to be significant in the p62 knockdown group based on p-value and z-score analysis, indicating its inhibition in this group (Fig. 5G). Additionally, analysis of the association between enriched cell functions and wound-healing pathways revealed important links, with FN1 and LAMC2 being key proteins within this pathway.

### Knockdown of p62 leads to increased cell apoptosis and inhibition of cell migration in HUVECs

**Fig. 6:**
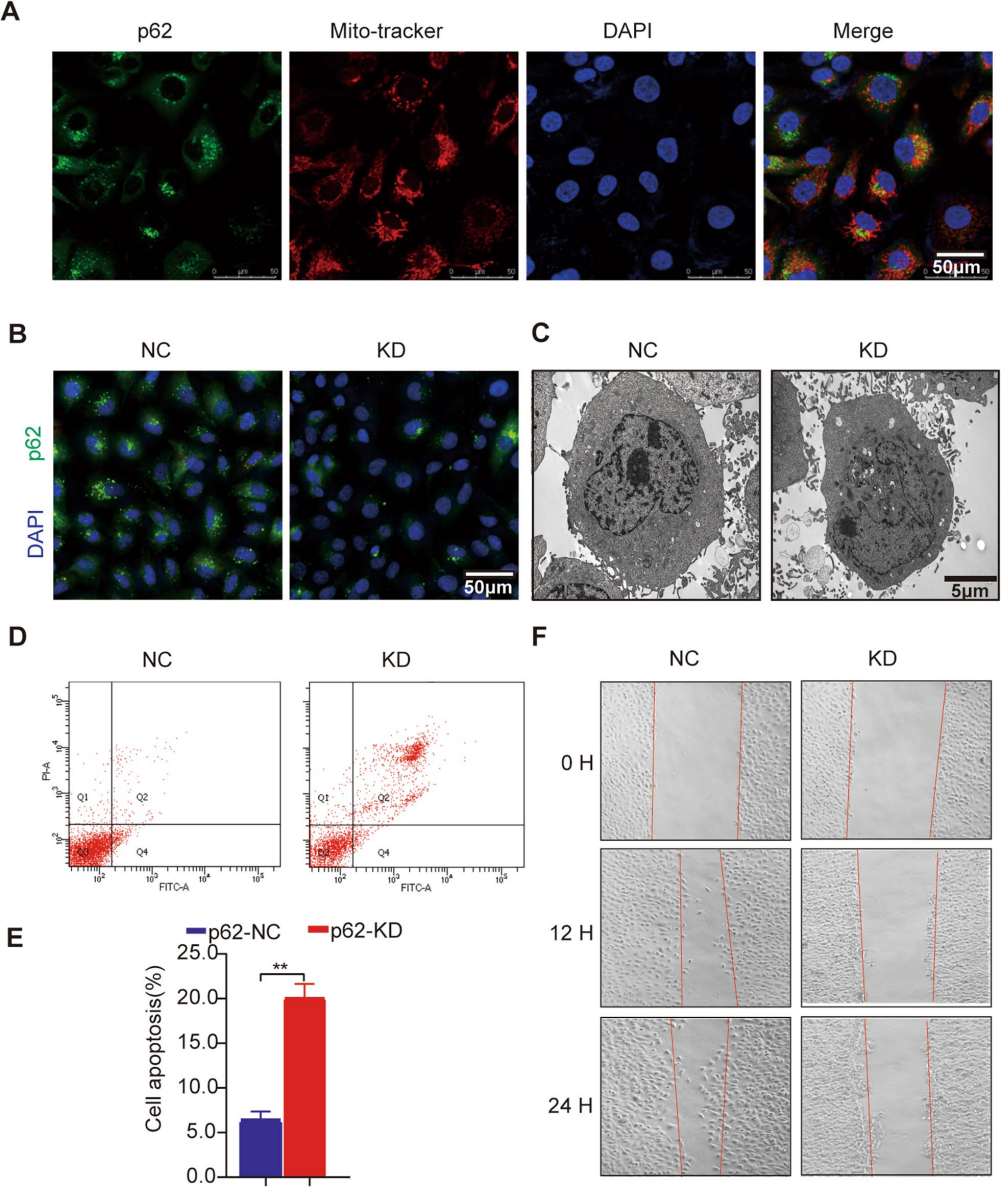
Knockdown of p62 in HUVEC increases cell apoptosis and inhibits migration. **A** Immunofluorescence detection of p62 localization in HUVEC. Scale bars = 50 μm. **B** Immunofluorescence showing validation of p62 (green) knockdown efficiency in HUVEC. DAPI dyed the nucleus blue. Scale bars = 50 μm. **C**. Electron microscopy image of HUVEC. Scale bars = 5 μm. **D** Flow cytometry analysis to detect apoptosis in HUVEC. **E** Quantitative analysis of apoptotic cells. **F** Wound healing assay to assess cell migration. ** *p* < 0.01

We investigated whether suppressing p62 could induce phenotype changes in HUVECs. Immunofluorescence analysis revealed that p62 is predominantly located in the cytoplasm of cells (Fig. 6A). We employed siRNA to knock down p62 (KD group) in HUVECs and validated the efficiency using immunofluorescence (Fig. 6B). Electron microscopy (EM) images showed a decrease in the number of mitochondria and disordered structure arrangement in the p62 siRNA group (Fig. 6C). Specific siRNA-mediated transfection of p62 in HUVECs resulted in increased apoptosis, as detected by flow cytometry (Fig. 6D, E). Moreover, p62 knockdown inhibited cell migration, as demonstrated by the wound healing assay (Fig. 6F) which was consistent with the major signal pathway results enriched by IPA.

### Inhibition of p62 in HUVECs increases fibrosis markers, including FN1, LAMC2, and EndoMT-related proteins

Endothelium-mesenchymal transition (EndoMT) is a crucial mechanism involved in the fibrosis process. Biomarkers of EndoMT include cytoskeletal proteins (α-SMA), cell surface proteins (E-cadherin), extracellular matrix proteins (collagens, FN1, and laminin), and specific transcription factors (SNAIL1, TWIST1, SLUG). During the EndoMT process, a fundamental change occurs with the loss of E-cadherin, followed by the activation of Vimentin and expression of FN1.

**Fig. 7:**
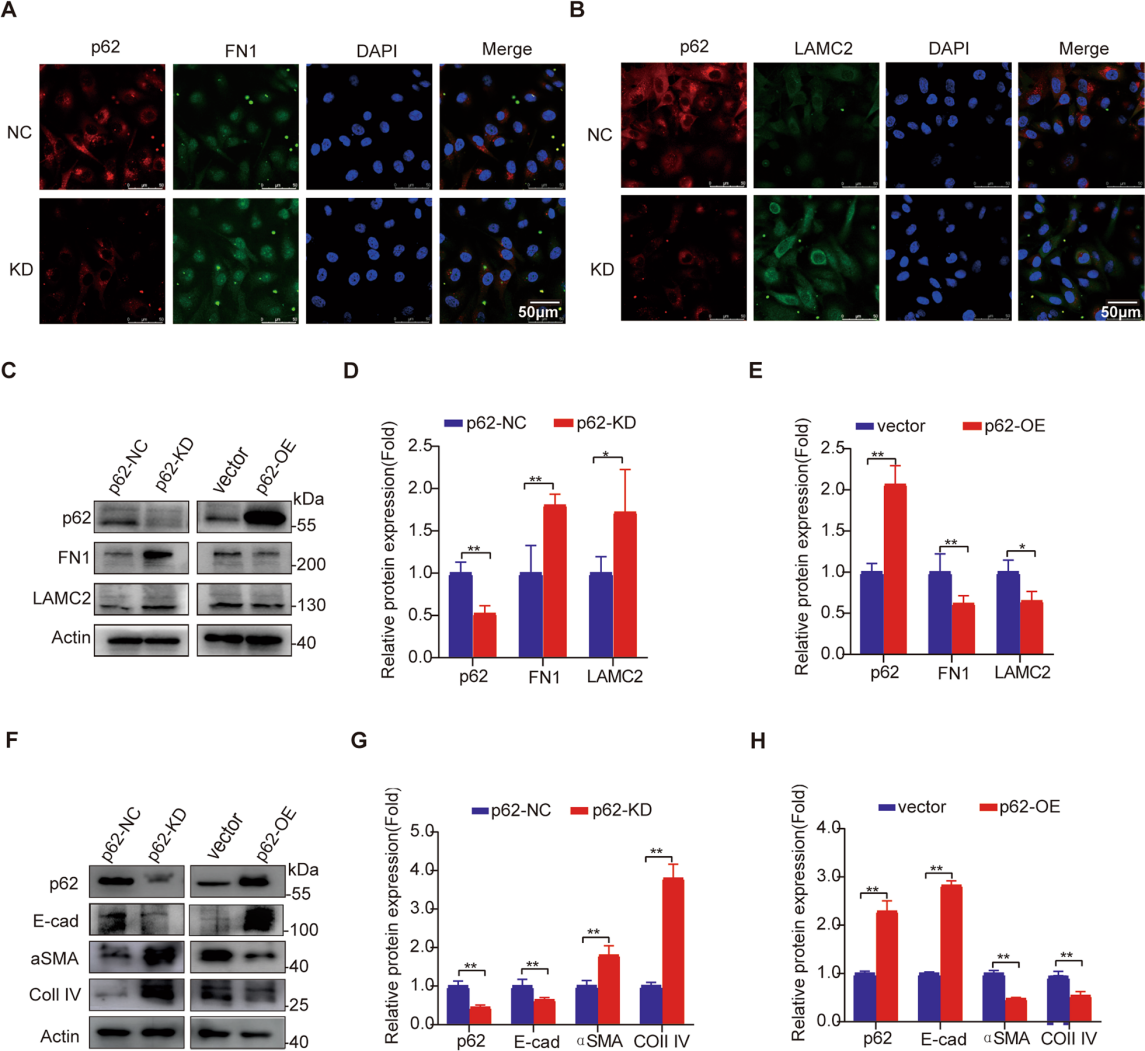
Knockdown of p62 in HUVEC significantly increases the expression of FN1, LAMC2, and other EndoMT markers. **A** Immunofluorescence detection of FN1 expression following p62 knockdown. Scale bars = 50 μm. **B** Immunofluorescence detection of LAMC2 expression following p62 knockdown. Scale bars = 50 μm. **C** Validation of FN1 and LAMC2 expression after transfection with p62siRNA or p62 overexpression plasmid using Western blot. **D** Semiquantitative analysis of FN1/LAMC2 in NC group and KD group at protein level. **E** Semiquantitative analysis of FN1/LAMC2 in Vector group and OE group. **F** Validation of endothelium mesenchymal transition (EndoMT) marker expression after transfection with p62siRNA or p62 overexpression plasmid using Western blot. **G** Semiquantitative analysis of EndoMT markers in NC group and KD group at protein level. **H** Semiquantitative analysis of EndoMT markers in Vector group and OE group. *p < 0.05 and ** *p* < 0.01

Using siRNA and plasmid overexpression, we performed transfections in HUVECs to manipulate p62 levels. Immunofluorescence staining revealed enhanced fluorescence of FN1 and LAMC2 after p62 knockdown, which was consistent with the results from protein mass spectrometry (Fig. 7A, B). Immunofluorescence results showed co-localization of p62 with FN1 or LAMC2, which mainly located in the cytoplasm. We further evaluated the protein levels of FN1 and LAMC2 in HUVECs using western blotting. We observed a significant increase in FN1 and LAMC2 protein levels following p62 siRNA interference (KD group) compared to the negative control (NC) group (Fig. 7C, D). Conversely, overexpression of p62 led to increased degradation of FN1 and LAMC2 (Fig. 7C, E) compared to the vector group (abbreviated as V). Additionally, we examined other EndoMT-related proteins in HUVECs using western blotting. Knockdown of p62 in HUVECs by siRNA resulted in elevated protein levels of α-SMA and collagen IV, as well as reduced levels of E-cadherin, indicating endothelium mesenchymal transition (Fig. 7F, G). Conversely, overexpression of p62 led to decreased levels of α-SMA and collagen IV, along with an increased level of E-cadherin, suggesting downregulation of EndoMT when p62 was sufficient (Fig. 7F, H). These findings indicate that p62 plays an essential role in endothelium homeostasis.

**Fig. 8:**
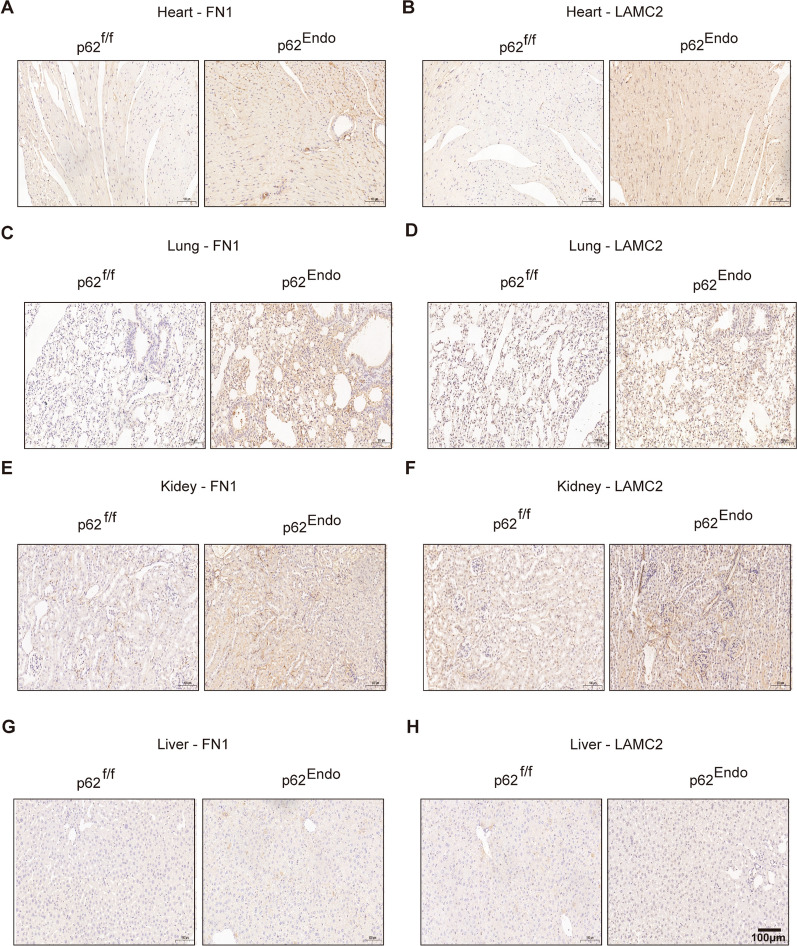
The knockout of p62 in endothelial cells results in the deposition of FN1 and LAMC2 proteins in heart, lung and kidney tissues. **A**, **B** IHC for FN1 and LAMC2 expression in the heart. **C**, **D**. IHC for FN1 and LAMC2 expression in lung tissue. **E**, **F** IHC for FN1 and LAMC2 expression in the kidney. **G**, **H** IHC for FN1 and LAMC2 expression in the liver. Scale bar = 100 μm

In order to further ascertain the expression of FN1 and LAMC2 in various organs of p62^Endo^ mice, we conducted IHC employing pertinent antibodies. The findings revealed a notable increase in the expression levels of FN1 and LAMC2 in the heart (Fig. 8A, B), lung (Fig. 8C, D) and kidney (Fig. 8E, F) tissues of p62 ^Endo^ mice compared to the control group. However, no significant differences were observed in the expression of FN1 and LAMC2 in liver tissues between the two groups (Fig. 8G, H), which aligns with the results obtained from Masson staining.

### p62 regulates FN1 and LAMC2 primarily through the autophagolysosomal pathway

**Fig. 9:**
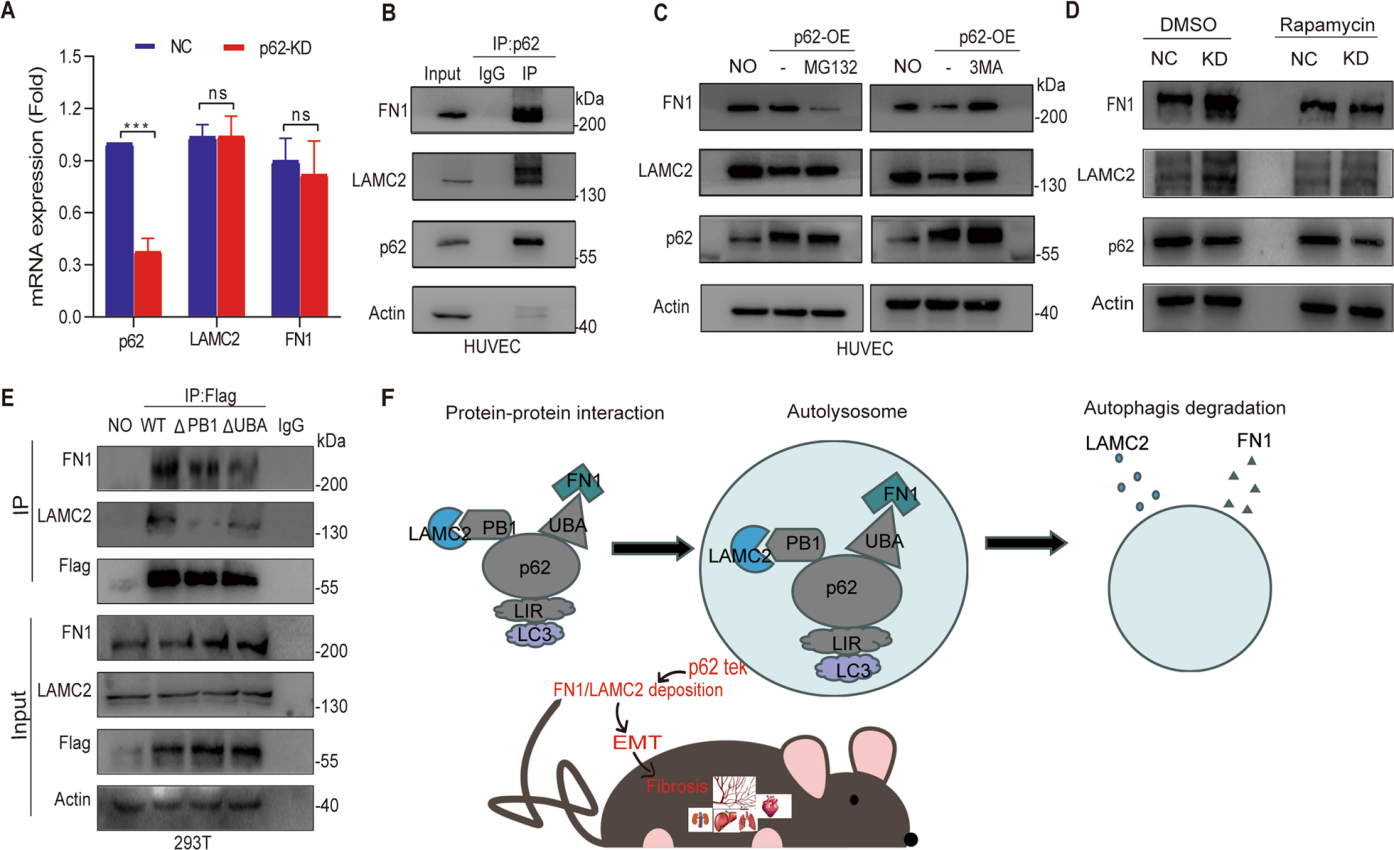
p62 interacts with FN1 and LAMC2 in endothelium cells, influencing their protein expression through the autophagy-lysosome pathway. **A** Quantitative real-time PCR (qRT-PCR) validation of FN1 and LAMC2 mRNA expression following p62 knockdown. **B** Immunoprecipitation (IP) assay confirming the interaction between p62 and FN1, as well as p62 and LAMC2. **C** Verification of FN1 and LAMC2 expression using the proteasome pathway inhibitor MG132 and the autophagy pathway inhibitor 3MA after p62 overexpression. **D** The autophagy inducer rapamycin exhibits potential in rescuing the accumulation of FN1 and LAMC2 caused by p62 knockdown. **E** Construction of truncated forms of p62 (PB1 and UBA), followed by IP to determine the binding regions of p62 with FN1 and LAMC2. **F** Mechanism diagram of tissue fibrosis caused by endothelial knockout in p62. ***p < 0.001

While the roles of FN1 and LAMC2 in fibrosis have been elucidated, the intracellular metabolism and degradation pathways of these proteins remain poorly understood. Since FN1 and LAMC2 are crucial indicators of the mesenchymal phenotype, it is essential to investigate their degradation pathways. Knockdown of p62 using siRNA resulted in the accumulation of FN1 and LAMC2, while their mRNA levels remained unchanged (Fig. 9A), suggesting that p62 may be involved in the protein degradation of FN1 and LAMC2. Furthermore, an immunoprecipitation assay using an anti-p62 antibody demonstrated that FN1 or LAMC2 could be pulled down by the anti-p62 antibody (Fig. 9B).

Autophagy and the proteasome system are two critical cellular pathways involved in protein degradation. Therefore, in the subsequent experiment, we aimed to determine whether the proteasome system or the autophagolysosomal pathway was responsible for the degradation of FN1 and LAMC2. Treatment with the proteasome inhibitor MG132 affected the levels of FN1 and LAMC2 but did not cause obvious aggregation of these proteins in HUVECs overexpressing p62 (Fig. 9C). In contrast, inhibition of autophagy using 3-methyladenine (3-MA) led to a decrease in the degradation of FN1 and LAMC2 (Fig. 9C). To verify whether autophagy activators could improve fibrosis caused by decreased p62, we conducted in vitro experiments whereby we downregulated p62 in HUVECs and treated the cells with the autophagy activator rapamycin. We found that under the influence of rapamycin, the levels of fibrotic markers such as FN1 or LAMC2 proteins significantly decreased compared with the DMSO intervention group with p62 siRNA (Fig. 9D), indicating that enhancing autophagy could ameliorate fibrosis induced by p62 knockdown. Concomitantly, we investigated the autophagic status in various tissues of p62 ^Endo^ mice. Interestingly, reduction in Beclin1 expression was observed in the heart (Additional file 1: Fig. S2A), lung (Additional file 1: Fig. S2B), and kidney (Additional file 1: Fig. S2C) tissues of p62^Endo^ mice compared to the control group, indicative of a manifestation of autophagy inhibition. Autophagy inhibition further contributed to the exacerbation of tissue fibrosis. Therefore, we have substantial grounds to speculate that autophagy inducers may have a potential ameliorative effect on fibrosis in p62^Endo^ mice, warranting further exploration in the future. Nevertheless, immunohistochemical analysis of Beclin1 in liver tissues of both groups of mice revealed mild upregulation in the expression levels of Beclin1 (Additional file 1: Fig. S2D), indicating no significant autophagy inhibition. This observation may be attributed to the absence of evident fibrosis in the liver tissues at present, hinting at a potential influence of p62 endothelial knockout on the autophagic status in different tissues. Furthermore, we cannot disregard the possibility of liver tissue fibrosis occurring in the coming months, and we shall continue to monitor it diligently.

To investigate the role of PB1 and UBA domains of p62 in the degradation of FN1 and LAMC2, we constructed plasmids with truncated PB1 (amino acids 21–85) and UBA (amino acids 385–440) domains based on the overexpression plasmid of full-length p62 with a flag tag. Antigen incubation with flag antibody working solution was performed, and immunoprecipitation experiments were conducted to detect the protein expression of FN1 and LAMC2. The results showed that compared to the overexpression plasmid group of full-length p62, the protein content of FN1 was significantly reduced or even undetectable in the UBA truncation group, suggesting the failure of interaction between p62 and FN1 after truncating the UBA domain (Fig. 9E). Similarly, the protein content of LAMC2 was significantly reduced or even undetectable in the PB1 truncation group, indicating the failure of interaction between p62 and LAMC2 after truncating the PB1 domain (Fig. 9E). These experimental findings demonstrate that p62 interacts with LAMC2 and FN1 respectively through its PB1 and UBA domains, mediating their degradation through the lysosomal autophagy pathway (Fig. 9F).

## Discussion

P62 plays a crucial role in maintaining homeostasis in various organs and cells through its protein–protein interaction domains and involvement in different physiological and pathological processes. In this study, we aimed to investigate the involvement of endothelium p62 in cardiac function and the function of other organs using mice with conditional knockout of p62 specifically in endothelium cells. Masson staining revealed significant fibrosis in multiple organs in the Tek-specific deletion group, indicating the impact of p62 on fibrotic processes. Our findings are consistent with a study conducted by Yuta Takagaki [21], where the knockout of Atg5 in endothelium cells resulted in similar phenotypes in mice.

Endothelium dysfunction is characterized by a shift from executing physiological functions to inflammation, adhesion, nitric oxide synthase uncoupling, oxidative stress, injury, and EndoMT [22]. EndoMT is a biochemical process that allows epithelial cells to acquire mesenchymal characteristics. Through protein mass spectrometry and IPA analysis, we have enriched fibrotic mechanisms associated with the upregulation of FN1 and LAMC2 following p62 knockdown.FN1 encodes fibronectin, a glycoprotein that exists as a soluble dimer in plasma and as a dimeric or multimeric form on the cell surface and extracellular matrix. Fibronectin is involved in various cellular processes, including cell adhesion, migration, embryogenesis [23], wound healing [24], host defense, and metastasis [25, 26]. LAMC2, an epithelial basement membrane protein, has been found to be significantly upregulated in various cancer cells, such as pancreatic cancer [27], lung adenocarcinoma [28], and esophageal squamous cell carcinoma [29]. LAMC2 promotes migration and invasion through EndoMT in lung adenocarcinoma. Both FN1 and LAMC2 play essential roles in extracellular matrix (ECM) maturation and its interaction with myofibroblasts, thereby promoting fibrosis. In tissues exhibiting significant fibrosis, we observed a notable increase in the levels of FN1 and LAMC2.They serve as markers of fibrosis [30–33].

p62 is a multifunctional protein that serves as a classical receptor for autophagy. It is distributed throughout the cell and participates in various signal transduction pathways. P62 mediates autophagy primarily through its Phox1 and Bem1p (PB1) domain, ubiquitin-association (UBA) domain, and LC3-interacting region (LIR). The PB1 domain of p62 plays a crucial role in binding with other PB1-containing proteins, serving as a scaffolding protein and facilitating p62 oligomerization [34]. Additionally, the PB1 domain is essential for p62 to bind with LC3, which acts as the autophagosome coat protein through the LIR motif [35]. The C-terminal UBA domain of p62 is responsible for binding to ubiquitinated proteins, targeting them for autophagic degradation via the LC3 motif [36]. These domains are vital for the degradation of numerous proteins, bridging the gap between the ubiquitin–proteasome system and autophagy, which are two distinct but interacting proteolytic systems [37, 38]. p62 also connects the autophagy pathway with the ubiquitin–proteasome system by interacting with Keap1. This interaction leads to the dissociation of Nrf2 from Keap1 and directs it towards autophagic degradation [39]. Studies have shown that p62-mediated autophagic degradation plays a role in the degradation of NOD2 [40]. The autophagic degradation of hexokinase 2 (HK2) has been implicated in regulating glycolysis through autophagy. The E3 ligase TRAF6 catalyzes Lys63-linked ubiquitination of HK2, which is critical for p62 to recognize and target HK2 for autophagic degradation [41]. In the context of head and neck squamous cell carcinoma (HNSCC), Liu [42] found that p62 regulates the degradation of FN1 through a p62-dependent autophagy-lysosome pathway. These findings highlight the role of p62 in facilitating autophagic degradation and its interaction with various proteins involved in different cellular processes.

In this study, we aimed to investigate whether p62 regulates EndoMT in fibrosis. We observed a significant increase in the protein levels of FN1 and LAMC2 after interfering with p62 using siRNA in HUVECs. Interestingly, the mRNA levels of FN1 and LAMC2 remained unchanged, suggesting that p62 might be involved in the stability of these proteins rather than affecting their expression at the transcriptional level. Further experiments in HUVECs revealed that overexpression of p62 led to increased degradation of FN1, while inhibition of autophagy using 3-methyladenine (3-MA) resulted in decreased degradation of FN1 and LAMC2. Moreover, the autophagy activator rapamycin has indeed demonstrated its ability to rescue the deposition of FN1 and LAMC2 resulting from p62 knockdown, thereby ameliorating EndoMT and fibrosis. Immunoprecipitation assays supported the involvement of p62 as an autophagy adapter in the autophagy-lysosome pathway responsible for the degradation of FN1 and LAMC2. Notably, other degradation pathways of EndoMT-related protein markers have been investigated. Twist is degraded by both autophagy and proteasome pathways [43], Snail undergoes natural degradation through autophagy [44, 45], and vimentin has been found to interact with p62, suggesting its association with autophagy pathways [46].

To elucidate the role of the PB1 domain and UBA domain in selective autophagy, immunoprecipitation assays were performed. The results revealed that deletion of the PB1 domain and UBA domain resulted in the failure of interaction between p62 and LAMC2 or FN1. Our results highlight FN1 and LAMC2 as markers of EndoMT with adverse effects on HUVECs. We elucidated the mechanism of autophagy-lysosome degradation of FN1 and LAMC2, demonstrating the involvement of p62 as an autophagy adapter that facilitates their degradation. The depletion of p62 in endothelial cells has been found to induce autophagy inhibition in multi organs. Firstly, it hampers the degradation of FN1 or LAMC2 by impeding their binding with p62 and affecting their autophagic pathways. Secondly, it exacerbates the accumulation of FN1 or LAMC2 by suppressing autophagy. Therefore, it is reasonable to speculate that autophagy inducers might alleviate the fibrosis caused by p62 depletion in endothelial cells. Furthermore, these autophagy inducers may have potential therapeutic effects on tissue fibrosis associated with autophagic dysregulation induced by other factors. Our study provides valuable insights into the role of p62 in maintaining endothelium homeostasis and its involvement in fibrotic processes and the PB1 domain or UBA domain indeed play a crucial role in selective autophagy.

## Conclusions

In conclusion, our present study provides clear evidence that deficiency of endothelium p62 plays a crucial role in cardiac diseases, including heart failure, and induces fibrosis in multiple organs. We identified FN1 and LAMC2 as important markers of EndoMT, which are degraded through the autophagy-lysosome pathway involving p62. Acting as an autophagy adapter protein, p62 facilitates the targeting of FN1 and LAMC2 to autophagosomes for intracellular degradation through PB1 or UBA domain. In this study, we made the novel discovery that the PB1 domain of p62 can interact with LAMC2, while the UBA domain binds to FN1. Therefore, targeting p62 as an intervention point could be valuable for further exploring the upstream and downstream relationships and post-translational modifications of p62. For example, designing small molecule compounds to stabilize p62 activity or its interaction with binding proteins, maintaining interaction stability, and reducing fibrotic marker deposition, could be one of the intervention strategies. Furthermore, utilizing autophagy activators to intervene in p62-deficiency-induced organ fibrosis or fibrosis caused by other factors holds promise in inhibiting fibrosis formation in vivo. This provides another important intervention strategy and lays the groundwork for future clinical translation for endothelium-related diseases.

### Supplementary Information

**Additional file 1: Figure S1.** No apparent abnormalities were observed in the cardiac function of the three-month-old p62 ^Endo^ mice. **Figure S2.** The knockout of p62 in endothelial cells leads to autophagy inhibition in multiple tissues.

## Data Availability

The data supporting the findings of this study could be obtained from the corresponding author.

## Abbreviations

FN1: Fibronectin1
LAMC2: Lamininγ2
EndoMT: Endothelium-to-mesenchymal transition
HUVEC: Human umbilical vein endothelium cells
3-MA: 3-Methyladenine
MRPL12: Mitochondrial ribosomal protein L12
TECs: Renal tubular epithelial cells
CKO: TEC-specific p62 conditional knockout
PB1: Phox1 and Bem1p domain
UBA: Ubiquitin-associated domain
LIR: LC3-interacting region
ECs: Endothelium cells
HE: Hematoxylin and eosin staining
BUN: Blood urea nitrogen
UA: Uric acid
ALT: Alanine aminotransferase
TG: Triglyceride
TCH: Total cholesterol
LDL: Low density lipoprotein
IPA: Ingenuity Pathway Analysis
IHC: Immunohistochemical staining
