# Supplementary material for: Endothelium-specific deletion of p62 causes organ fibrosis and cardiac dysfunction
Source: J Transl Med. 2024 Feb 16;22:161. doi: 10.1186/s12967-024-04946-w (PMC10870664; doi:10.1186/s12967-024-04946-w)

**Figure S1. No apparent abnormalities were observed in the cardiac function of the three-month-old p62 ^Endo^ mice.**

A. Echocardiogram of the three-month-old mice from parasternal short axis view. B. Masson's staining of the myocardium in the three-month-old mice. Scale bar=200μm.C. Masson's staining of lung tissue in the three-month-old mice. Scale bar=50μm.


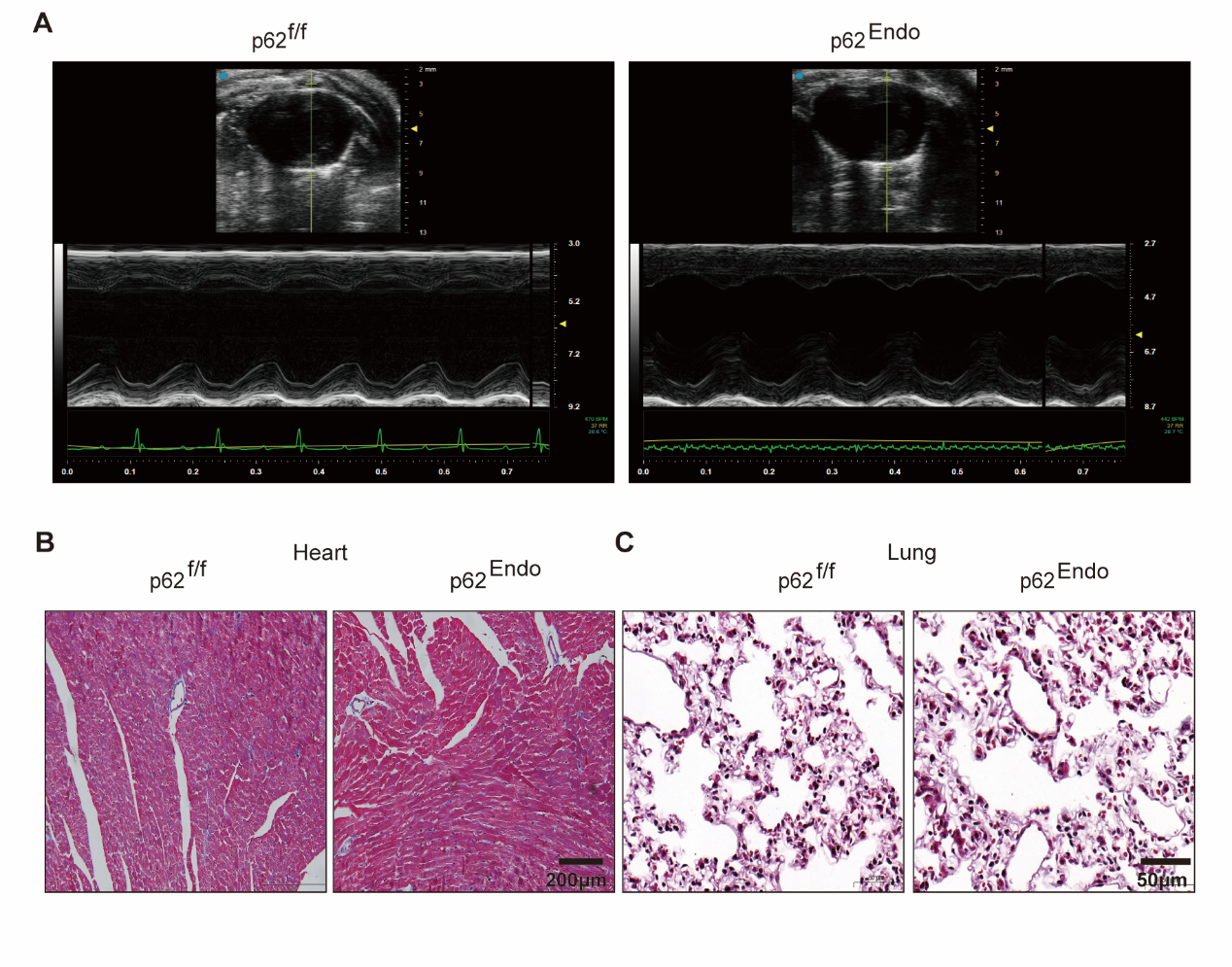


**Figure S2. The knockout of p62 in endothelial cells leads to autophagy inhibition in multiple tissues.**

A. IHC for Beclin1 expression in the heart.

B. IHC for Beclin1 expression in lung tissue.

C. IHC for Beclin1 expression in the kidney.

D. IHC for Beclin1 expression in the liver. Scale bar = 50μm.


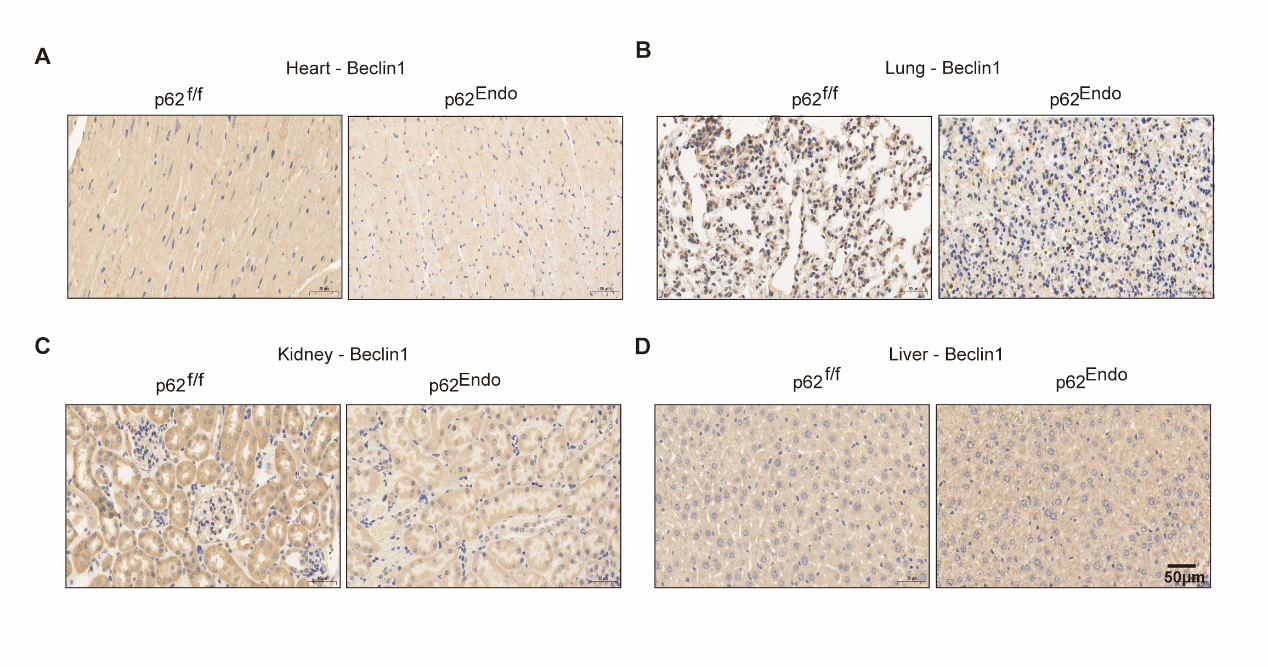

Supplement: Supplementary file 1 — Additional file 1: Figure S1. No apparent abnormalities were observed in the cardiac function of the three-month-old p62 Endo mice. Figure S2. The knockout of p62 in endothelial cells leads to autophagy inhibition in multiple tissues. [file 12967_2024_4946_MOESM1_ESM.docx]
